# Supplementary material for: CellSNAP: a fast, accurate algorithm for 3D cell segmentation in quantitative phase imaging
Source: J Biomed Opt. 2024 Apr 18;29(Suppl 2):S22706. doi: 10.1117/1.JBO.29.S2.S22706 (PMC11025678; doi:10.1117/1.JBO.29.S2.S22706)
Supplement: Supplementary file 1 [file JBO_029_S22706_SD001.pdf]

CellSNAP: A fast, accurate algorithm for 3D cell segmentation in quantitative phase imaging

Piyush Raj [1], Santosh Kumar Paidi [1], Lauren Conway [2], Arnab Chatterjee [1], Ishan Barman [1,3,4]

1. *Department of Mechanical Engineering, Johns Hopkins University, Baltimore, Maryland, USA*
2. *Department of Chemical and Biomolecular Engineering, Johns Hopkins University, Baltimore, Maryland, USA*
3. *The Russell H. Morgan Department of Radiology and Radiological Science, The Johns Hopkins University, School of Medicine, Baltimore, Maryland, USA*
4. *Department of Oncology, Johns Hopkins University, Baltimore, Maryland, USA*

Corresponding author: Ishan Barman – ibarman@jhu.edu

Supplementary Information

Intersection over Union:

Intersection over Union is defined as the ratio of overlap area to total area.

$$IoU = \frac{A \cap B}{A \cup B}$$

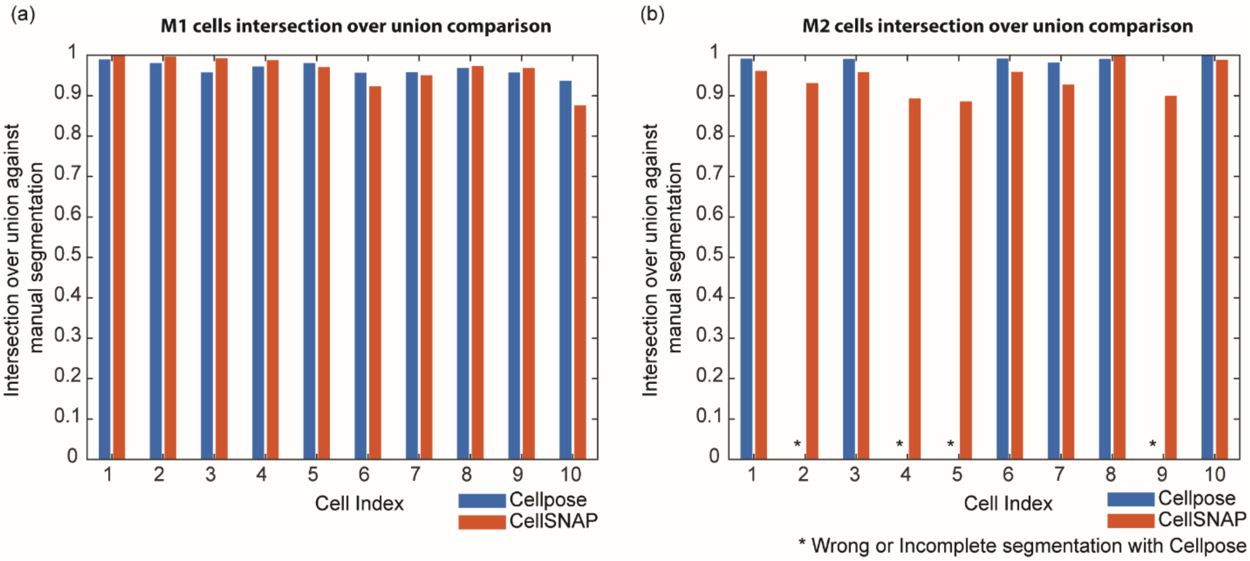

Figure S1: *Intersection over union comparison against the manual annotated and segmented data (a) M1 cells and (b) M2 cells for Cellpose and CellSNAP algorithm.*

Intersection over Union (IoU) is a popular metric in the field of computer vision to assess and evaluate the accuracy of object detection and segmentation tasks [26] [27]. It quantifies the overlap between the predicted segmentation and the ground truth, offering a clear measure of performance that is both simple and effective. The metric calculates the percentage of overlap, which is easy to understand and interpret. It ensures that both the detection of relevant areas and the avoidance of

irrelevant areas are factored into the evaluation. By measuring the overlap, IoU inherently penalizes both false positives and false negatives, providing a holistic assessment of the model's performance.

Based on the IoU metric, Cellpose and CellSNAP perform similarly in M1 cells, while CellSNAP outperforms Cellpose in M2 cells.

While IoU is scale-invariant, its performance can be disproportionately affected by small objects or thin structures due to their smaller area contributing less to the overall score [28]. To address this shortcoming, we have also used the Dice coefficient as a comparison metric.

Dice Coefficient

The dice coefficient is defined as the ratio of twice the intersection of two segmentations divided by the sum of individual segmentations.

$$Dice\ Coeffecient = \frac{2 * (A \cap B)}{|A| + |B|}$$

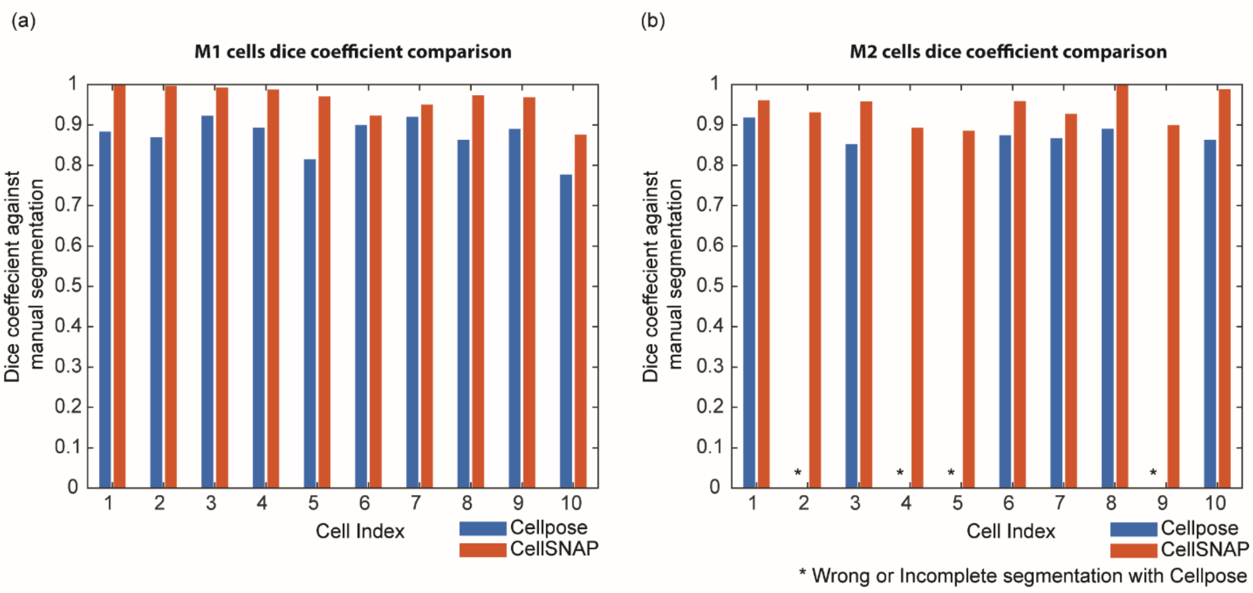

Figure S2: *Dice coefficient comparison against the manual annotated and segmented data (a) M1 cells and (b) M2 cells for Cellpose and CellSNAP algorithm.*

The Dice coefficient, also known as the Sørensen–Dice index or Dice similarity coefficient (DSC), is another metric used for comparing the similarity between two samples. It is particularly popular in the field of medical image segmentation because of its sensitivity to small structure [29] [30]. The Dice coefficient tends to give more weight to the correctly identified pixels, which can be particularly useful in medical imaging where small structures (where background is much bigger than segmented image) are important.

Based on dice coefficient values, CellSNAP outperforms Cellpose for both M1 and M2 cells.

Hausdorff distance

Hausdorff distance is defined as the maximum distance out of the set of data of minimum distances between the contours of two segmentations.

$$Hausdorff\ distance = \max\big(h(A,B), h(B,A)\big)$$

where h is nearest point in other set

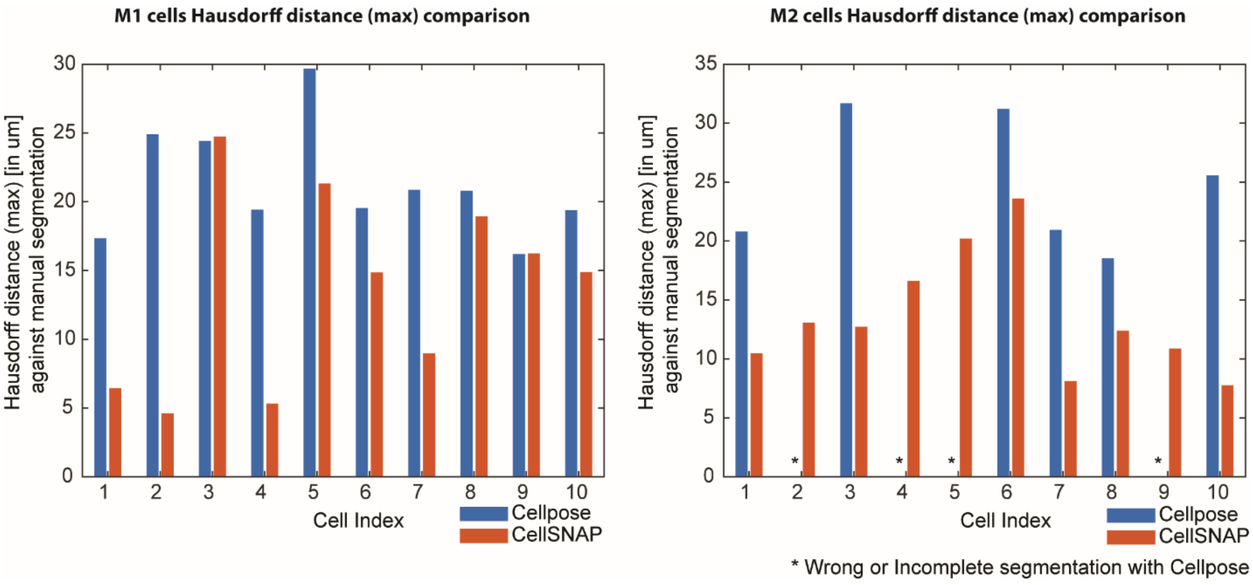

Figure S3: *Hausdorff distance comparison against the manual annotated and segmented data (a) M1 cells and (b) M2 cells for Cellpose and CellSNAP algorithm.*

While IoU and Dice coefficients are standard metrics to evaluate segmentation accuracy, both these metrics are insufficient when it comes to geometric accuracy in terms of shape of segmented object. By measuring the maximum distance from a point in one set to the closest point in another set, Hausdorff distance metric is particularly useful for assessing the spatial accuracy of boundary predictions [31].

Based on Hausdorff distance, CellSNAP outperforms Cellpose for both M1 and M2 cells.

While Hausdorff distance is best suited for applications where the exactness of the boundary delineation is critical, it can be very sensitive to outliers and noise. Therefore, combining this metric with IoU and dice coefficient allows for a comprehensive evaluation of segmentation quality.

Taking all these three metrics into consideration, we can conclude that CellSNAP outperforms Cellpose for both M1 and M2 cells.
